# Bibliometric review on sleep and Alzheimer disease between 1986 and 2023

**DOI:** 10.1097/MD.0000000000035764

**Published:** 2023-11-03

**Authors:** Xiaoyu Sun, Chao He, Huiling Qu

**Affiliations:** a Department of Neurology, General Hospital of Northern Theater Command, Shenyang, China.

**Keywords:** Alzheimer disease, bibliometric, CiteSpace, sleep

## Abstract

**Objective:**

Alzheimer disease (AD) is a major disease that affects the elderly worldwide. Therefore, this study aimed to examine the relationship between AD and sleep disorders, identify journal publications and collaborators, and analyze keywords and research trends using a bibliometric method.

**Methods:**

Data retrieval is based on the Web of Science Core Collection database. CiteSpace V.6.1.R6 was used to analyze bibliometric analysis, calculate centrality, and draw co-occurrence maps of countries/regions, institutions, authors, published journals, cited literature, keyword co-occurrence maps, cluster maps, time graphs, and emergent maps from January 1986 to April 2023.

**Results:**

There were 4677 publications relevant to AD and sleep disorders. From 1986 to 2023, the number of publications per year showed an increasing trend. The United States not only has the largest output of publications, the first in the centrality ranking, but also owns the 3 highest frequencies of publication institutions. The journal NEUROLOGY has the highest citation frequency, reaching 2671, with a median centrality value of 0.64. A comprehensive analysis of centrality showed that AD, circadian rhythm, dementia, Parkinson disease, sleep, and older adults are both high-frequency words and high centrality words, becoming core keywords in this field.

**Conclusions:**

This was the first study to provide an overview, about the current main status of development, hot spots of the study, and the future trends in sleep disorders and AD, which provides a comprehensive review of the trends and gaps in field of sleep and AD, and thus lays the groundwork for future research.

## 1. Introduction

Sleep is crucial for the physiology of the central nervous system.^[1,2]^ Changes in sleep quality and structure can occur throughout a lifetime and worsen during healthy aging.^[3–5]^ Several studies have shown that sleep is associated with central nervous system diseases, such as cerebrovascular diseases, Parkinson disease (PD), multiple sclerosis, epilepsy, headache, pain, especially neurodegenerative diseases^.[6,7]^ Alzheimer disease (AD) is the most important cause of dementia, accounting for nearly 60% to 80% of all dementia cases in the world.^[8]^ Sleep disorders are common symptoms associated with AD.^[9]^ More than 60% of AD patients experience sleep disorders, which typically occur in the early stages of the disease and even before severe cognitive decline.^[10]^ Various epidemiological studies have shown a correlation between sleep disorders and an increased risk of AD or AD related pathology.^[11]^ Recent studies suggest that sleep disorders not only occur before typical cognitive impairment episodes, but are also related to the pathogenesis of AD, which may have a decisive impact on symptoms and course.^[12]^ These latest findings suggest that sleep disorders are a potential modifiable risk factor for AD and may be a new target for disease modification therapy to prevent the development of AD and/or improve cognitive decline in AD patients.^[13]^ Therefore, sleep disorders deserve more attention in research, diagnosis, and treatment. Understanding this interaction is crucial as it will increase people’s understanding of the role of sleep in the development of AD and the impact of AD on sleep quality.^[14]^ This may provide clues on how sleep interferes with the cognitive and behavioral symptoms of dementia. In addition, understanding the relationship between AD and sleep can provide a deeper understanding of the pathophysiological mechanisms of AD^.[15]^ It is important to track the progress and contributions of clinical doctors and basic scientists in order to improve diagnosis and better treatment. In this study, we used bibliometric methods to summarize recent, highly cited studies, the countries with the greatest contributions, hot spots, and published work over the past decade on the relationship between sleep disorders and AD. Bibliometric analysis is a scientific method that utilizes quantitative statistics to construct a research topic co-occurrence network.^[16]^ Bibliometric analysis is a tool that allows for statistical examination of scientific publications. They mainly help identify past and future trends in certain research topics.^[17]^ Some reviews on sleep disorders and AD have been published; however, a comprehensive and visualized analysis remains lacking.^[11,14,18–24]^ Therefore, we conducted a bibliometric analysis of sleep disorders and AD research to reveal the dynamic developments in this field. This study contributes to a comprehensive understanding of this topic and guides future research directions.

## 2. Methods

### 2.1. Search strategy

**Figure 1.**
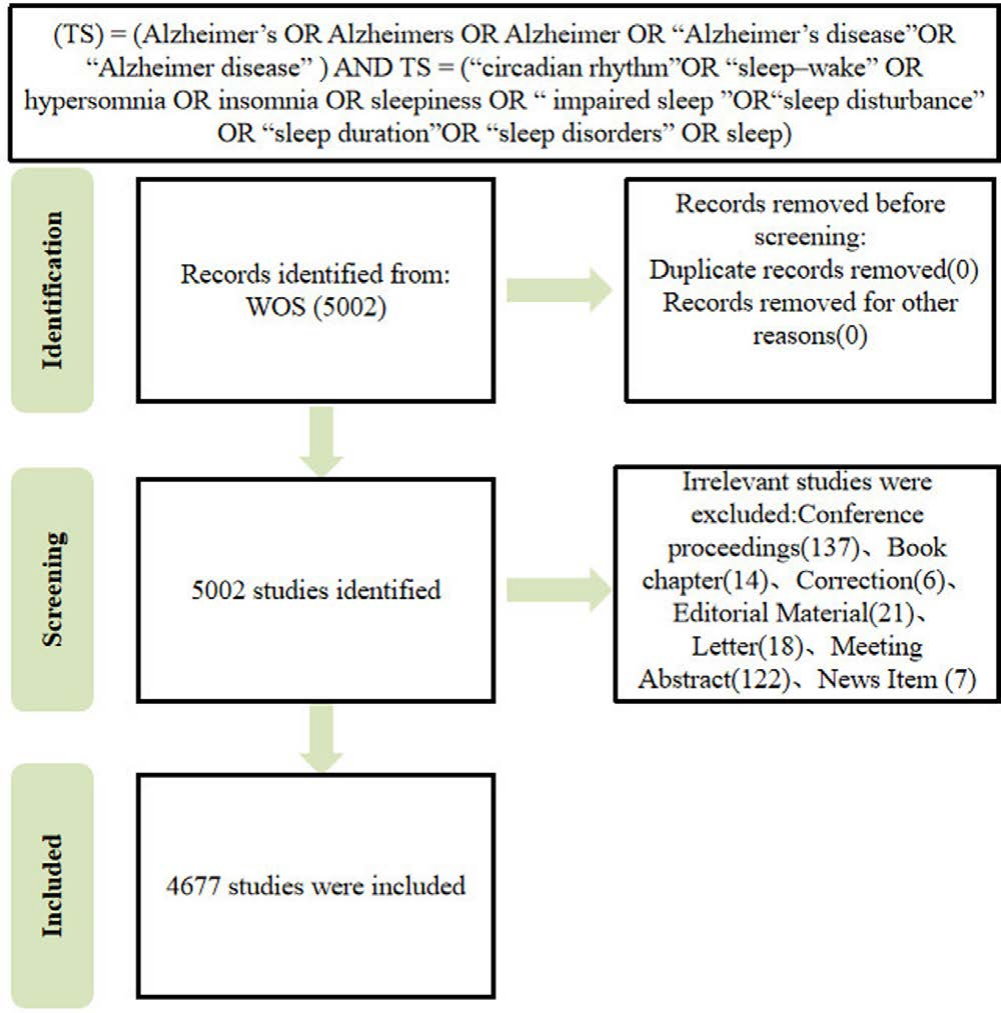
Flow diagram of the publications screening process.

Data retrieval is based on the Web of Science Core Collection (WoSCC) database. WOSCC is a standardized online database, which is considered the most suitable database for bibliometric analysis. To ensure the accuracy of the data, the literature search was completed in 1 day (April 20, 2023), to January 1,1986, with April 20,2023, selected as the time frame for this study. All articles from the search were preliminarily included, and we screened all articles by reading the title, abstract, and author keywords. The types of studies included original articles, reviews, and conference papers, conference reviews, and letters, and English was the primary language. Articles were not officially published; conference abstracts and proceedings and corrigendum documents; unrelated articles were excluded. In order to identify any possible differences in selection, 2 researchers independently searched the raw data. A PRISMA 2020 flow diagram^[25]^ showing the detailed filtering process through the different phases of the analysis can be found in Figure 1. After multiple attempts to search using different keyword combinations, our final search strategy was to use Boolean operator tools such as “OR” and “AND” to combine with keywords related to “sleep” and “Alzheimer disease,” limiting the title search. The search terms were as follows: (TS) = (Alzheimer’s OR Alzheimers OR Alzheimer OR “Alzheimer’s disease” OR “Alzheimer disease”) AND TS = (“circadian rhythm”OR “sleep–wake” OR hypersomnia OR insomnia OR sleepiness OR “impaired sleep ” OR “sleep disturbance” OR “sleep duration” OR “sleep disorders” OR sleep). Ultimately, a total of 5002 unique articles were included, imported into bibliometrics, and transferred into CiteSpace software for bibliometric analysis. The retrieval strategy used in this study is shown in Figure 1 and Table 1.

**Table 1:** Detailed search strategy.

The search terms	(TS) = (Alzheimer’s OR Alzheimers OR Alzheimer OR “Alzheimer’s disease” OR “Alzheimer disease”) AND TS = (“circadian rhythm” OR “sleep–wake” OR hypersomnia OR insomnia OR sleepiness OR “impaired sleep” OR “sleep disturbance” OR “sleep duration” OR “sleep disorders” OR sleep)
5002 studies identified	Paper 3558, Review paper 1119, Conference proceedings (137), Book chapter (14), Correction (6), Editorial Material (21), Letter (18), Meeting Abstract (122), News Item (7)
4677 studies were included	Paper 3558, Review paper 1119

### 2.2. Analysis methods and tools

After importing the literature data from the WOS data download into Cite Space V.6.1.R6, deduplication was performed first. After running the deduplication program, there were no duplicates in the literature included in this study, and the original data could be used directly for subsequent analysis. CiteSpace was used to analyze bibliometric analysis, calculate centrality, and draw co-occurrence maps of countries/regions, institutions, authors, published journals, cited literature, keyword co-occurrence maps, cluster maps, time graphs, and emergent maps. Parameters were set as follows: time slice, January 1986 to April 2023, 1 year per slice. Select “Pruning,” “Pathfinder,” and “Pruning network,” and save the default settings for other options. Select g index 20 (k = 20) to analyze keyword co-occurrence, clustering, timeline, time zone map, and prominence map; Select Top 20 to analyze the co cited images of authors, institutions, and journals. In the visualization diagram, nodes and font size represent the frequency, lines (edges) between nodes represent the relationships between nodes, the thickness of lines represents the degree of tightness of relationships, and the color of nodes and lines represents the distance between years.

## 3. Results

### 3.1. Publication trends

**Figure 2.**
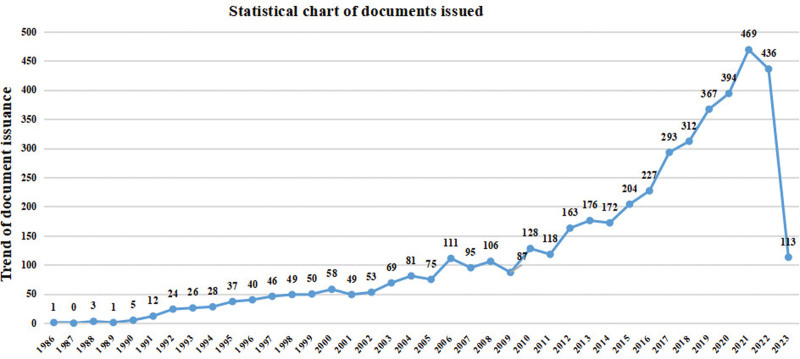
Annual number and growth trend of publications from 1986 to 2023.

Over the past year from January 1986 to April 2023, there were 4677 publications relevant to AD and sleep disorders, with a mean of 129.92 publications per year. From 1986 to 2023, the number of publications per year showed an increasing trend (Fig. 2). From Figure 2, it can be seen that from 1986 to 1990, the field received low attention and had a small number of publications, with a total of 10 articles published in the past 4 years. From 1991 to 2005, the overall trend of publications in this field was on the rise, with a flat overall growth. In 2006, the annual publication volume exceeded 100 for the first time and then slowly increased. By 2015, the annual publication volume had exceeded 200 for the first time and increased year by year, reaching a peak of 469 articles in 2021, marking an explosive period for research in this field. In recent years, the number of publications in this field has also maintained a high level. Generally, with the increasing attention to sleep disorders, there has been a surge of interest in the relationship between sleep disorders and AD from both annual publications and citation numbers.

### 3.2. Bibliometric analysis of countries/regions, institutions, journals and author

#### 3.2.1. Analysis of countries.

**Figure 3.**
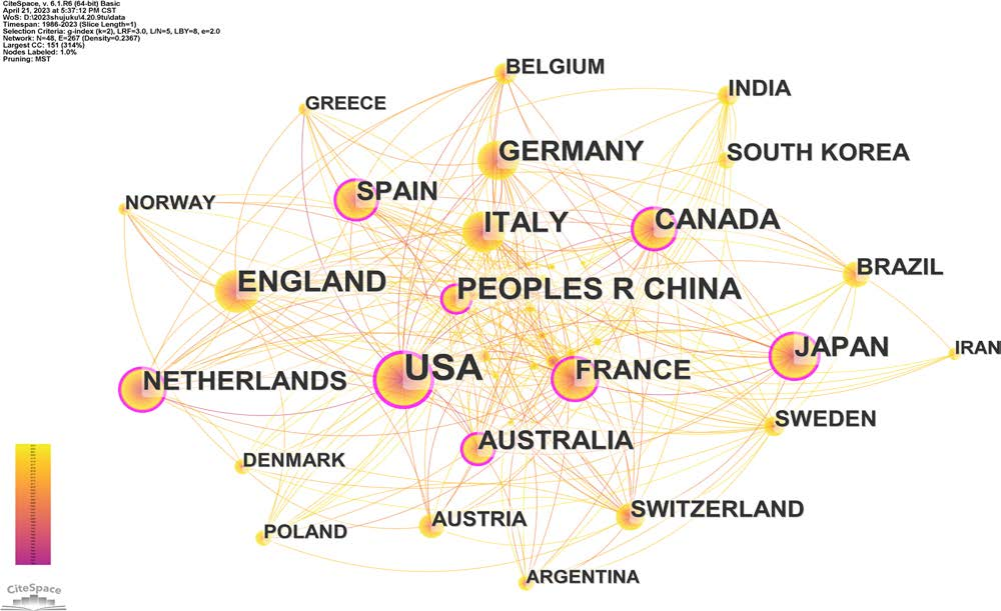
The map of co-countries. The nodes represent countries, the number of lines represents the intensity of cooperation between countries.

The different colors in the center of the nodes in Figure 3 reflect the differences in the timing of the first publication in different countries. Among them, the center of the node is dark orange, indicating that the country’s first collaborative publication was earlier, while the center of the node is bright yellow, indicating that the country’s first collaborative publication was later. The information of the top 10 countries in terms of publication time is shown in Table 2. As can be seen in Figure 3, the country with the largest node and the highest frequency of publications is the United States, and the publications have reached 1772 times since 1990, followed by CHINA (427) since 2001, ENGLAND (381) since 1995, ITALY (324) since 1992, CANADA (301) since 1992, and GERMANY (275) since 1993, JAPAN (254) since 1991, FRANCE (238) since 1986, SPAIN (201) since 1997, NETHERLANDS (188) since 1988, AUSTRALIA (186) since 1993, SOUTH KOREA (119) since 2011, and SWITZERLAND (100) since 2000. It can be clearly seen from the figure that the United States is at the core of the network, and we can see that there are a total of 48 countries and 267 contacts, which means there is a lack of communication with some countries. Therefore, attention should be paid to cooperation between countries to promote development in this field.

**Table 2:** Top 10 countries/regions in frequency/centrality of publications.

Rank	Frequency	Centrality	Year	Country
1	238	0.1	1986	FRANCE
2	188	0.11	1988	NETHERLANDS
3	1772	0.31	1990	USA
4	61	0.02	1990	AUSTRIA
5	254	0.13	1991	JAPAN
6	35	0.01	1991	GREECE
7	30	0.01	1991	ISRAEL
8	324	0.09	1992	ITALY
9	301	0.15	1992	CANADA
10	23	0.01	1992	FINLAND

#### 3.2.2. Analysis of institutions.

**Figure 4.**
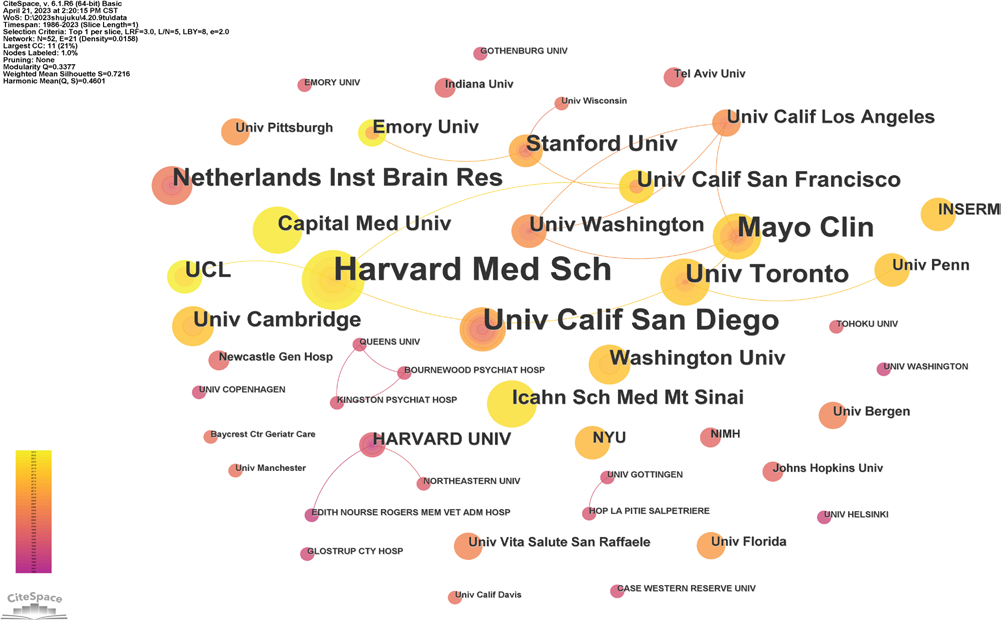
The map of co-institutions. The nodes represent institutes, the number of lines represents the intensity of cooperation between institutions.

The graph analysis of publishing institutions can detect their academic contributions to the field, as well as the cooperation between institutions. Figure 4 clearly shows the cooperation between institutions. A total of 52 institutions had published articles on AD and sleep disorders. The publishing institutions are mainly concentrated in universities, and some belong to research organizations. The organization with the highest frequency of posting 73 times was Harvard Med Such, followed by Univ California San Diego, with 42 posts and Mayo Clin with 38 posts. Other institutions are ranked according to their frequency of publication as follows: Harvard Univ 30 times, Univ Toronto 25 times, Netherlands Inst Brain Res 24 times, Stanford Univ 16 times, Univ California San Francisco and UCL 15 times, Univ Cambridge 14 times, Washington Univ, Capital Med Univ, Univ Washington 13 times, Icahn Sch Med Mt Sinai 12 times.

In addition, nodes with multiple connections represent a significant contribution and influence of the institution in the academic network. This influence is quantified as the centrality of a node, which refers to the number of shortest paths that pass through the nodes in the network, and is a measure of the size of the connection role played by the nodes in the overall network. Among them, Harvard Med Sch has the highest intermediary centrality. The top 10 intermediaries with centrality are ranked in Table 3.

**Table 3:** Top 10 institutions in frequency/centrality of publications.

Rank	Frequency	Centrality	Year	Institution
1	73	0.05	2017	Harvard Med Sch
2	25	0.04	2008	Univ Toronto
3	38	0.03	2006	Mayo Clin
4	16	0.03	2001	Stanford Univ
5	15	0.03	2009	Univ Calif San Francisco
6	42	0	1998	Univ Calif San Diego
7	30	0	2001	Harvard Univ
8	24	0	1998	Netherlands Inst Brain Res
9	15	0	2020	UCL
10	14	0	2014	Univ Cambridge

#### 3.2.3. Analysis of journals.

**Figure 5.**
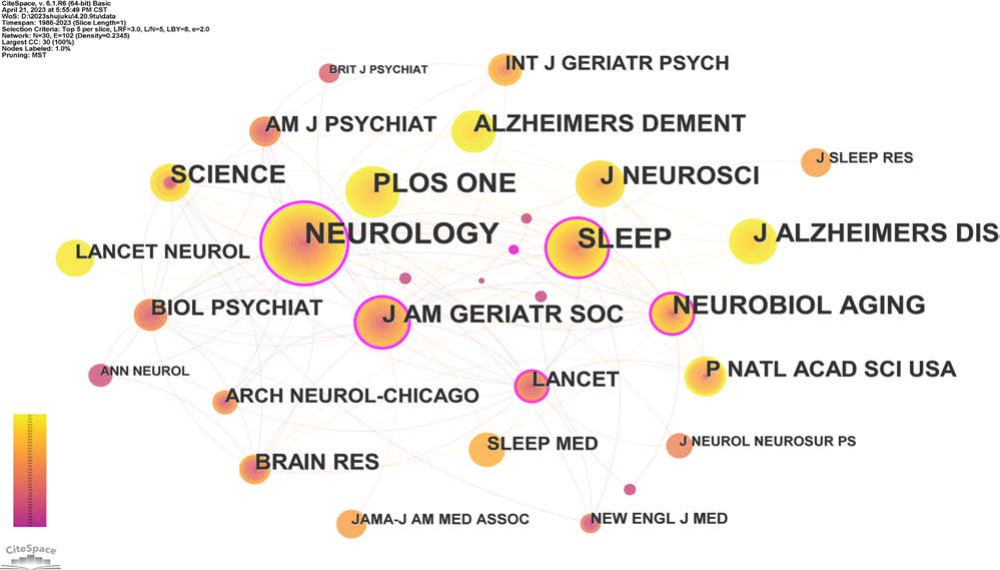
The co-occurrence map of published journal.

The journal NEUROLOGY has the highest citation frequency, reaching 2671, with a median centrality value of 0.64, indicating that the journal contains the most articles and has the greatest impact (Fig. 5). The journal SLEEP ranks second, with a citation frequency of 1999 and a median centrality value of 0.29. The journal PLOS ONE ranks third with a citation frequency of 1337, and the journal J ALZHEIMERS DIS has a citation frequency of 1040, which is also the most frequently cited article. The other journals with citation frequencies below 1000 are ranked in order of citation frequency: NEUROBIOL AGING, J NEUROSCI, SCIENCE, J AM GERIATR SOC, ALZHEIMERS DEMENT, P NATL ACAD SCI USA, BRAIN RES, BIOL PSYCHIAT, AM J PSYCHIAT, LANCET, ARCH NEUROL CHICAGO, LANCET NEUROL, NT J GERIATR PSYCH, ISLEEP MED, NEW ENGL J MED, JAMA-J AM MED D ASSOC, J SLEEP RES, J NEUROL NEUROSUR PS ANN NEUROL, BRIT J PSYCHIAT, etc. The information of the top 10 journals with descending citation frequency is shown in Table 4.

**Table 4:** Top 10 journals in frequency/centrality of publications.

Rank	Citations	Centrality	Year	Journal
1	2671	0.64	1990	NEUROLOGY
2	1999	0.29	1990	SLEEP
3	1337	0.01	2014	PLOS ONE
4	1040	0.03	2018	J ALZHEIMERS DIS
5	985	0.39	1990	NEUROBIOL AGING
6	855	0.01	2005	J NEUROSCI
7	854	0.03	1992	SCIENCE
8	733	0.42	1991	J AM GERIATR SOC
9	616	0	2019	ALZHEIMERS DEMENT
10	359	0.01	1998	P NATL ACAD SCI USA

#### 3.2.4. Analysis of authors.

**Figure 6.**
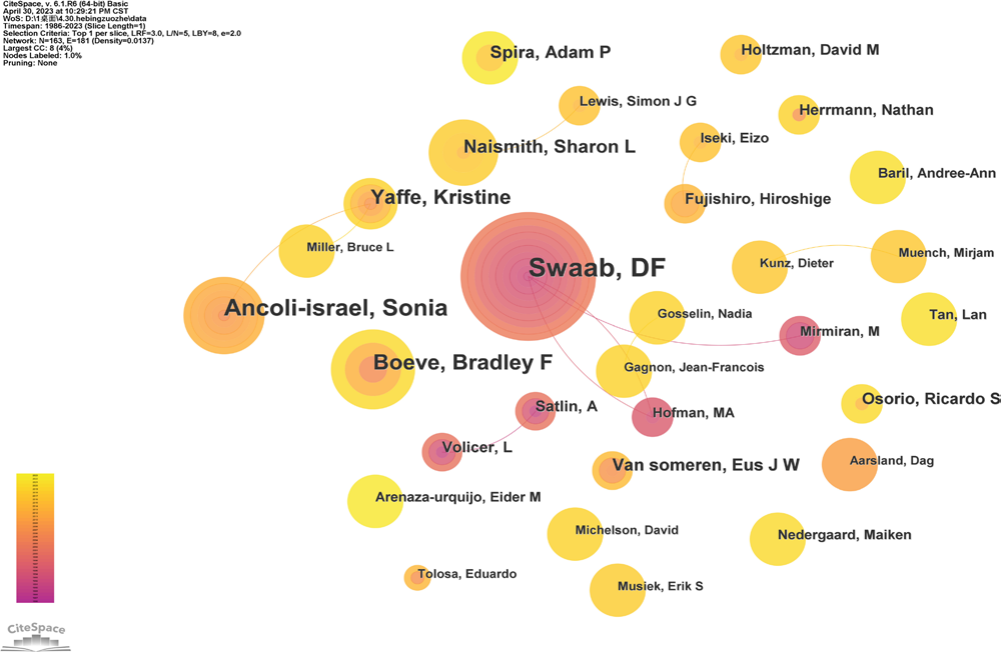
The map of coauthors. The nodes in the map represent coauthors, and lines between the nodes represent co-citation relationships.

There are 163 authors who have contributed to sleep and AD research in the WOSCC. The researchers and their collaborations regarding sleep and AD research are shown in Figure 6. In terms of the number of publications, the 3 most influential authors are Ancoli-israel, S(n = 37); Swaab, DF(n = 34); Boeve, Bradley F(n = 17). Ancoli-israel S had a significantly higher number of citations than the other authors. We can see that the collaboration was not very close, and scholars need to strengthen cooperation in the future.

### 3.3. Research topic analysis

#### 3.3.1. Analysis of co-occurrence keywords.

In this study, there were a total of 128 keywords, and the number of their co-occurrences reached 447. Table 5 shows that the top five keywords are alzheimers disease (n = 2835), dementia (n = 724), parkinsons disease (n = 543), circadian rhythm (n = 502), and mild cognitive impairment (n = 465). The greater the centrality value, the more cooperation between the node and other nodes. Table 6 shows that Alzheimers disease (centrality: 0.62), circadian rhythm (centrality: 0.24), dementia (centrality:0.17), Parkinsons disease (centrality:0.15) and senile dementia (centrality:0.14) had a high degree of centrality during this period. A comprehensive analysis of centrality showed that AD, circadian rhythm, dementia, PD, sleep, and older adults are both high-frequency words and high centrality words, becoming core keywords in this field.

**Table 5:** Top 10 keywords in cited times.

Rank	Citations	Centrality	Year	Keyword
1	2835	0.62	1991	alzheimers disease
2	724	0.17	1992	dementia
3	543	0.15	1995	parkinsons disease
4	502	0.24	1991	circadian rhythm
5	465	0.08	2005	mild cognitive impairment
6	322	0.11	1992	sleep
7	300	0.09	2009	older adult
8	288	0.06	1997	disturbance
9	269	0.07	2006	risk
10	244	0.03	2000	cognitive impairment

**Table 6:** Top 10 keywords in centrality.

Rank	Citations	Centrality	Year	Keyword
1	2835	0.62	1991	alzheimers disease
2	502	0.24	1991	circadian rhythm
3	724	0.17	1992	dementia
4	543	0.15	1995	parkinsons disease
5	68	0.14	1991	senile dementia
6	148	0.12	1994	cerebrospinal fluid
7	322	0.11	1992	sleep
8	300	0.09	2009	older adult
9	202	0.09	1992	diagnosis
10	142	0.09	1995	rem sleep

**Figure 7.**
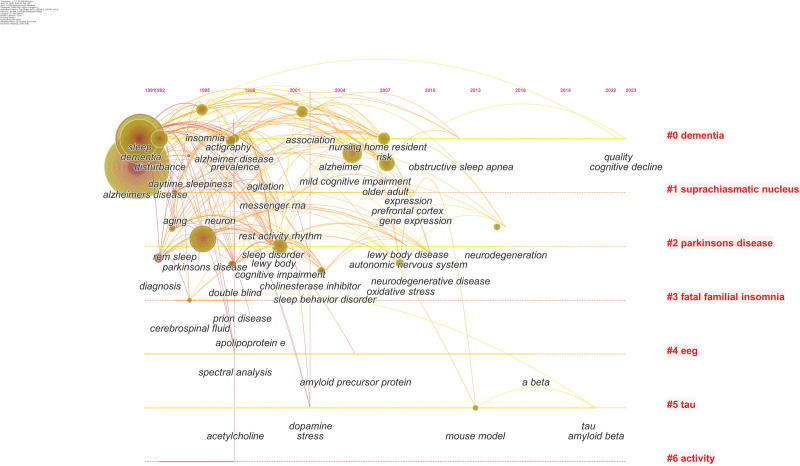
The timeline viewer of keywords cluster.

Based on keyword co-occurrence, CiteSpace can use the logarithmic likelihood ratio algorithm for clustering analysis of keywords. Keyword clustering is a network of related keywords in research fields similar to research topics, with each cluster identified by frequently appearing titles in publications. Cluster numbering starts from # 0; the smaller the numbers, the more keywords they contain. Based on keyword clustering analysis, 7 clusters were formed, from which we extracted these 7 clusters for timeline analysis (Fig. 7). The clusters include #0 dementia, #1 suprachiasmatic nucleus (SCN), #2 parkinsons disease, #3 fatal familial insomnia, #4eeg, #5 tau, #6 activity. Papers in larger clusters could spread across half of the timeline, such as cluster #0, #1, #2, #3.

#### 3.3.2. Future research direction analysis.

**Figure 8.**
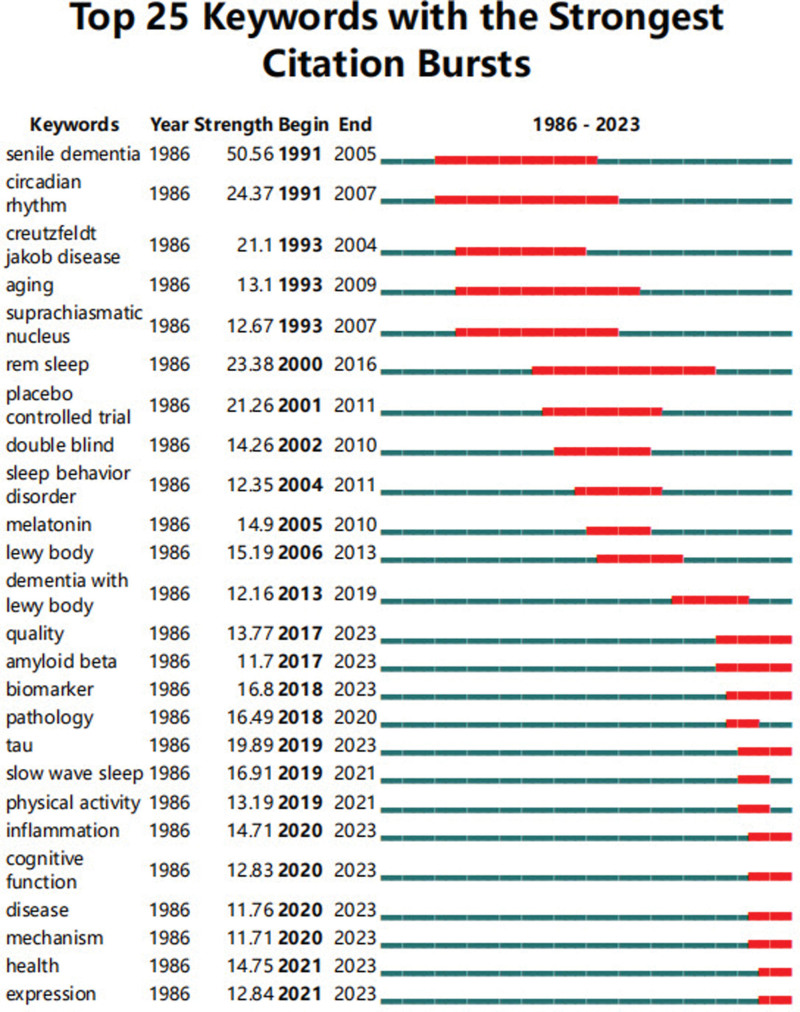
The emergent map of keywords. The red horizontal stripes represent the years with the most frequent keywords. The green horizontal stripes represent the years with the most infrequent keywords.

Keywords burst detection was conducted to track hot topics and research trends. The time span of the theme evolution is from 1986 to 2023 (Fig. 8). The blue line represents the time interval, while the red line represents the duration of the citation burst. It is recommended to track and observe the evolution of hot topics. The hot topics switched from senile dementia, circadian rhythm, Creutzfeldt Jakob disease, aging, SCN, rem sleep, placebo controlled trial, double blind, sleep behavior disorder to tau, slow wave sleep, physical activity, inflammation, cognitive function, disease, mechanism, health, and expression. Some topics burst with long durations, “senile dementia” and “ circadian rhythm” emerged in 1991 and lasted for a long time, making them early hot keywords. In addition, other words that appeared earlier and had a longer duration, such as “creutzfeldt Jakob disease,” “aging,” and “superparachiasmatic nucleus,” also appeared earlier. On the other hand, based on the keyword emergence intensity indicated by “Strength,” “senile dementia” ranks first with an intensity index of 50.56; other keywords with significant prominence include circadian rhythm, Creutzfeldt Jakob disease, rem sleep, placebo controlled trial, tau, etc. These hot words have also become “strong hotspots” in academic tracking.

## 4. Discussion

Research on sleep disorders and AD has drawn an increasing amount of attention over the past years. This study used bibliometrics and visual analysis methods to discuss the research trends and hot spots in sleep disorders and AD research field from 1986 to 2023. In the case of sleep disorders and AD, a significant number of scientific publications have been published lately. According to this study, sleep disorders and AD research has shown an increasing trend over the past years, with 2021 being the year with the most published literature. Our study found that research on the relationship between sleep disorders and AD began in 1986, and the number of published papers is increasing every year. The number of published papers in 2021 is 3.66 times that of 2010 and 8.09 times that of 2000. Although there was a slight decrease compared to 2021, the number of publications remained relatively high in 2023.

Among the top 10 countries in terms of publication time, the United States not only has the largest output of publications, the first in the centrality ranking, but also owns the 3 highest frequency of publication institutions—Harvard Med Sch, Univ Calif San Diego and Mayo Clin. Similarly, Harvard Med Such from the United States also has the top centrality. It is worth noting that among the top 6 most frequently cited authors, 4 of them were from the United States and Ancoli-israel, S with the highest number of publications was also from the United States indicating that the United States and its institutions have played a leading role in sleep disorders and AD research. Although the United States is leading in this field and the frequency of publication is more than 5 times that of Canada, which is ranked second, interestingly, Swaab, DF from the Netherlands Institute for Neuroscience in Netherlands was the secondary influential and contributing author in this field. He began studying the effects of sleep disorders and AD in 1992, which made a certain contribution to the knowledge foundation in this field. Hence, it can be concluded that, in addition to the United States mentioned above, Netherlands also plays an indispensable role in this field. Inter-institutional cooperation shows regionalization, with relatively few international multicenter cooperations. We can also see that the cooperation between these authors is not very close. International cooperation of the authors should be strengthened and more meaningful research could be carried out in the future. These findings enable authors to identify leading research institutions and authors in the field, in order to seek better academic cooperation.

Through the analysis of journals and the most highly cited studies, we found that NEUROLOGY (2671 citations), Q1, and 2021 JCR impact factor of 12.2577 has paid the most attention to this field. NEUROLOGY is the leading clinical neurology journal worldwide, and we believe that these innovative and groundbreaking high-quality research results confirm the importance of sleep for AD patients and actively promote research in the field of AD. NEUROLOGY is the official journal of the American Academy of Neurology indicating the leading role of the United States in this field. In addition, it is worth noting that there are a relatively large numbers of papers in this field. SLEEP (1999 citations), PLOS ONE (1337 citations), and J ALZHEIMERS DIS (1040 citations) all have been cited more than 1000 times. This shows that these journals identified in Table 3 are mainly regarding neurology, SLEEP, and AD, which is related to AD and its pathogenic mechanism.

Co-occurrence keywords analysis is commonly used to identify research hotspots. Keywords analysis showed that “alzheimers disease,” “circadian rhythm,” “dementia,” “parkinsons disease,” had high influential. It should be noted that, at present, researchers have made many advancements in sleep disturbances in AD and PD. AD and PD are the 2 most common neurodegenerative diseases in the older adults, both of which exhibit sleep disorders, including insomnia, drowsiness, and excessive daytime naps. The molecular basis of sleep disorders in AD and PD patients may involve damage to the hypothalamus and brainstem nuclei that control the circadian rhythm. Disturbance of neurotransmitter and hormone signal transduction (such as serotonin, norepinephrine, and melatonin) and neurotrophin BDNF may lead to disease process.^[26]^ Sleep disorders are often observed in AD and PD patients, not only as accompanying symptoms, but also as precursor symptoms before the onset of these diseases.^[27]^ According to the cluster analysis results, studies related to “suprachiasmatic nucleus” have attracted wide attention. The SCN is the main circadian rhythm pacemaker, and some changes may occur during disease. The deterioration of its function may play a crucial role in the relationship between the pathophysiology of AD and the development of sleep abnormalities. So sleep abnormalities have generally been considered merely a consequence of AD pathology.^[28]^ Another interestingly cluster was “tau” which has been a research hotspot and with the strongest citation bursts began in 2019. β-Amyloid (Aβ) and tau were the 2 main proteins accumulate in the brain in AD.^[29]^ Sleep seems to have a direct protective effect on a key protein that drives AD pathology, and long-term sleep deprivation can rapidly increase tau protein within hours and promote the spread of tau protein pathology in mice and human brains. These results indicate that tau can be considered as a neoplasm marker of sleep disorder in AD pathology.^[30]^ “Slow wave sleep” has gradually attracted attention since 2019. Recent research results show that the cognitive decline and pathophysiology of AD are closely related to the interruption of slow wave sleep, and slow wave sleep is the deepest stage of sleep. Slow wave sleep is crucial for memory function and displays a potentially causal and bidirectional link to the accumulation of Aβ.^[31]^ AD patients spend less time during non-rapid eye movement sleep and exhibit a decrease in slow wave activity. Consistent with the key role of slow wave sleep in memory consolidation, a decrease in slow wave activity is associated with impaired memory consolidation in AD patients. The destruction of slow wave activity leads to further accumulation of Aβ and tau in AD.^[32]^ We propose that therapeutic targeting of slow wave activity in AD might lead to an effective treatment for Alzheimer patients. Thematic evolution analysis further indicates that researchers have made many advancements in AD physical activity field since 2019. Sleep and physical activity may have a synergistic effect in preventing AD.^[33]^ More exercise can lead to better sleep quality, which itself is an important protective factor against AD.^[34]^ People with good sleep quality are more likely to exercise. Together, they create a powerful barrier against dementia. A comprehensive approach to reducing various risk factors in AD may be more successful than a single approach.^[35]^We found that inflammation is the research frontier since 2020, indicating that inflammation has aroused great interest since 2020. Sleep is regulated by circadian rhythm and homeostasis, so pathological inflammation may destroy the chemical signals needed to maintain healthy sleep. This way, the activation of the immune system can affect sleep physiology. On the contrary, sleep disorders can exacerbate symptoms or increase the risk of inflammation/neurodegenerative diseases.^[36]^

### 4.1. Limitations

When interpreting the results of this study, some scientific factors should be considered. Firstly, these publications are only sourced from the WOS core collection database and do not record references to textbooks or other databases. Secondly, our analysis only includes English publications, which may result in incomplete searches. Furthermore, new publications may subsequently be added to the 2023 queue, but the database was not updated at the time of retrieval. However, this part of the literature is very small and will not cause a large error in the analysis.

## 5. Conclusion

To the best of our knowledge, this was the first study to provide an overview about the current main status of development, hot spots of study, and the future trends in sleep disorders and AD. We identified the 4677 most cited papers in field of sleep and AD, and the research results clearly indicate a surge in interest in sleep disorders related AD research. In summary, the present study provides a comprehensive review of the trends and gaps in the field of sleep and AD, and thus lays the groundwork for future research.

## Author contributions

**Conceptualization:** Huiling Qu.

**Writing – original draft:** Xiaoyu Sun.

**Writing – review & editing:** Chao He.
